# Genome Sequences of Nine Gram-Negative Vaginal Bacterial Isolates

**DOI:** 10.1128/genomeA.00889-16

**Published:** 2016-09-29

**Authors:** Grace E. Deitzler, Maria J. Ruiz, Wendy Lu, Cory Weimer, SoEun Park, Lloyd S. Robinson, Kymberlie Hallsworth-Pepin, Aye Wollam, Makedonka Mitreva, Warren G. Lewis, Amanda L. Lewis

**Affiliations:** a Department of Molecular Microbiology, Washington University School of Medicine, St. Louis, Missouri, USA; b Department of Obstetrics and Gynecology, Washington University School of Medicine, St. Louis, Missouri, USA; c Department of Internal Medicine, Washington University School of Medicine, St. Louis, Missouri, USA; d Center for Women’s Infectious Disease Research, Washington University School of Medicine, St. Louis, Missouri, USA; e McDonnell Genome Institute, Washington University School of Medicine, St. Louis, Missouri, USA

## Abstract

The vagina is home to a wide variety of bacteria that have great potential to impact human health. Here, we announce reference strains (now available through BEI Resources) and draft genome sequences for 9 Gram-negative vaginal isolates from the taxa *Citrobacter, Klebsiella, Fusobacterium, Proteus*, and *Prevotella.*

## GENOME ANNOUNCEMENT

Reproductive and urinary tract infections are a major cause of morbidity and mortality for women worldwide (1, 2). Bacterial vaginosis (BV) is an imbalance of the vaginal microbiota that is associated with higher risks of sexually transmitted infections, urinary tract infections, and poor health outcomes among pregnant women (3–10). Women with BV have few lactic acid-producing bacteria (lactobacilli) and high levels of fastidious anaerobic bacteria. A variety of species within the *Bacteroidetes* and *Fusobacteriales* (among other taxa) have been isolated from women with BV, often from sites in the upper reproductive tract (e.g., placenta and amniotic fluid) (5, 11–14). Despite the widespread health complications associated with BV, its etiology is poorly characterized, and current treatment options are often met with recurrences (15).

Urinary tract infection (UTI) is another recurrent urogenital condition that is common among women and associated with poor pregnancy outcomes (1). *Escherichia coli* is the most common cause of UTI (16), and there are many dozens of available isolates and genomes of *E. coli* available for study. *Citrobacter* and *Klebsiella* spp. are less common etiologic agents of UTI. It is thought that the vagina can sometimes act a reservoir for uropathogens; however, few vaginal isolates of uropathogenic bacterial species are available as fully sequenced deposited isolates. The lack of reference strains and corresponding reference genomes of urogenital bacteria hinders research progress aimed at understanding how bacteria cause infection in the genital and urinary tracts. Here, we present annotated genome sequences of nine Gram-negative vaginal isolates, which have been made available to the research community through BEI Resources.

Vaginal swabs were collected from nonpregnant and pregnant women according to Washington University institutional review board (IRB)-approved protocols 201108155 and 201103082. Anaerobic vaginal swabs from reproductive-age pregnant and nonpregnant women were streaked onto agar medium and cultivated anaerobically. A detailed description of the isolation of these bacteria will be provided elsewhere.

Genomes were assembled using the One Button Velvet (1.1.06) pipeline (17), with hash sizes of 31, 33, and 35 after downsizing the input data to 100× coverage. Postassembly, we set the minimum length for contigs to 200 bp, ran an internal core gene screen on the assembly (as defined by the Human Microbiome Project [HMP] [18]), removed adapters, trimmed low-quality regions, and screened for contamination. The gene annotation process included generating both *ab initio* and evidence-based (BLAST) predictions. Coding sequences were identified using GeneMark and Glimmer3 (19, 20). Loci were then defined by clustering predictions with the same reading frame. We evaluated predictions using the NR and Pfam databases (21) and resolved overlaps between adjacent coding genes. Intergenic regions not spanned by GeneMark and Glimmer3 were subjected to a BLAST search against NCBI’s nonredundant (NR) database, and predictions were generated based on protein alignments. tRNA genes and noncoding RNA genes were found using tRNAscan-SE, RNAmmer, and Rfam (22–24). The final gene set was annotated for metabolic pathway predictions using KEGG (25), subcellular localization using PSORTb (26), and functional domain associations using InterProScan (27).

### Accession number(s).

Nucleotide sequences have been deposited in GenBank under the accession numbers listed in Table 1. The sequences described in this paper are the first versions. We have also made the strains available to the research community by depositing them with the Biodefense and Emerging Infections (BEI) Research Resource Repository (see BEI numbers in Table 1).

**TABLE 1:** Strain names and accession numbers

Species	Strain name	BEI catalog no.	Nucleotide sequence accession no.
*Citrobacter freundii*	GED7749C	HMS-1280	LRPR00000000
*Citrobacter koseri*	GED7778C	HMS-1288	LRPS00000000
*Fusobacterium* sp.	CMW8396	HMS-1274	LRPX00000000
*Fusobacterium nucleatum*	MJR7757B	HMS-1289	LRPY00000000
*Klebsiella pneumoniae*	MJR8396D	HMS-1265	LRQC00000000
*Prevotella bivia*	GED7880	HMS-1270	LTAG00000000
*Prevotella bivia*	GED7760C	HMS-1286	LRQF00000000
*Prevotella corporis*	MJR7716	HMS-1294	LRQG00000000
*Proteus mirabilis*	GED7834	HMS-1271	LSGS00000000
